# Design and evaluation of carbon fabric/epoxy reinforced with reduced graphene oxide (RGO) hybrid composite for gamma ray shielding

**DOI:** 10.1039/d5ra06722g

**Published:** 2025-11-04

**Authors:** Summan Urooge, Ahsan Irshad, Danish Arif, Srosh Fazil, Khurram Liaqat, Akif Safeen, Basit Ali

**Affiliations:** a Department of Chemistry, University of Poonch Rawalakot 12350 Pakistan khurramliaqat@upr.edu.pk; b Department of Physics, University of Poonch Rawalakot 12350 Pakistan akifsafeen@upr.edu.pk; c Department of Chemistry and Materials Science, School of Chemical Engineering, Aalto University P.O. Box 16100, FI-00076 Aalto Finland basit.ali@aalto.fi

## Abstract

This study presents a comprehensive investigation into the gamma-ray shielding effectiveness and structural characterization of epoxy/carbon fabric composites reinforced with reduced graphene oxide (RGO) nanoparticles. A series of composite samples were fabricated with varying RGO content (0–5 wt%), and their attenuation capabilities were evaluated through experimental and computational analysis. Key radiation shielding parameters, including Mass Attenuation Coefficient (MAC), Linear Attenuation Coefficient (LAC), Half Value Layer (HVL), Tenth-Value Layer (TVL), and Mean Free Path (MFP), were determined for each sample using standard gamma sources (Cs-137, Co-60, and Ba-133) and validated against theoretical models *via* Phy-X/PSD and XCOM software. Structural and morphological characterization was performed using X-ray Diffraction (XRD), Fourier Transform Infrared (FTIR) Spectroscopy, and Scanning Electron Microscopy (SEM). Results indicated a significant enhancement in attenuation performance with increasing RGO content. Notably, sample 3, containing 5 wt% RGO, exhibited the highest attenuation efficiency, attributed to improved particle dispersion and increased density. XRD patterns confirmed successful integration of RGO within the epoxy matrix, while FTIR spectra revealed characteristic functional groups supporting chemical interactions between RGO and the polymer network. SEM analysis further demonstrated a well-bonded and homogeneously distributed filler network, contributing to enhanced barrier properties. These findings affirm the potential of epoxy/carbon fabric RGO hybrid composites as promising candidates for lightweight radiation shielding applications.

## Abstract 2

A carbon fabric/epoxy RGO hybrid composite exhibits enhanced gamma-ray shielding through synergistic nanoscale absorption and macroscale attenuation, outperforming conventional GO/polymer systems.

## Graphical abstract

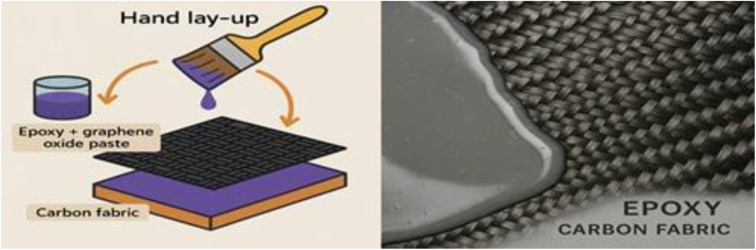

## 1 Introduction

The rapid advancement of nuclear technologies, space exploration, and modern medical imaging has intensified the demand for innovative radiation shielding materials that are both efficient and environmentally sustainable. Conventional shielding materials such as lead (Pb) provide excellent attenuation owing to their high atomic number (Z) and density; however, their inherent toxicity, brittleness, and high weight impose significant limitations for contemporary applications, particularly in wearable devices, aerospace systems, and flexible electronics.^1–3^ This has created an urgent demand for advanced alternatives that combine radiation shielding efficiency with mechanical robustness, lightweight characteristics, and environmental safety.^4–6^ Polymer matrix composites (PMCs) have emerged as promising candidates for such multifunctional applications due to their tunable properties, low cost, and ease of processing.^7^ Among these, epoxy resins are widely used owing to their mechanical strength, thermal stability, and chemical resistance. Nevertheless, pristine epoxy exhibits poor gamma attenuation because it is primarily composed of low-Z elements (C, H, O). To overcome this limitation, the incorporation of high-Z fillers and nanostructured reinforcements has been extensively explored to enhance radiation shielding without compromising mechanical integrity.^8–10^

Graphene oxide (GO), a two-dimensional derivative of graphite, has recently attracted attention in this context. Its high surface area, layered morphology, and abundance of oxygen-containing functional groups enable homogeneous dispersion in polymer matrices, improve interfacial bonding, and provide additional pathways for photon interaction through scattering, absorption, and electron generation.^11–13^ Several studies have demonstrated that GO-based epoxy nanocomposites exhibit enhanced attenuation efficiency against ionizing radiation, making them promising candidates for lightweight shielding systems.^14,15^ To further improve performance, hybrid composites combining GO-reinforced epoxy matrices with carbon fabric layers have been proposed. Carbon fabrics act as structural reinforcements, significantly enhancing tensile strength and modulus while contributing to radiation attenuation *via* π electron interactions and secondary scattering effects.^16–20^ This hybrid design not only improves load transfer and crack resistance but also increases the effective interaction volume for incoming photons through the synergistic action of 2D nanofillers and 3D woven fabrics.^21–24^

Alongside experimental efforts, theoretical modeling tools such as Phy-X/PSD and WinXCom are widely used to predict shielding parameters, including the mass attenuation coefficient (MAC), linear attenuation coefficient (LAC), half value layer (HVL), mean free path (MFP), and tenth value layer (TVL) across a broad photon energy spectrum.^25–27^ These tools enable rapid screening of material compositions and support the design of composites tailored for specific radiation environments. In this study, we report the design, fabrication, and evaluation of carbon fabric/epoxy hybrid composites reinforced with GO for gamma-ray shielding. Experimental measurements were conducted using Ba-133, Co-60, and Cs-137 gamma sources, while theoretical simulations were performed with Phy-X and WinXCom to compute shielding parameters over a wide energy range. The novelty of this work lies in the development of a hybrid epoxy composite reinforced with both GO nanofillers and woven carbon fabric. Unlike most polymer/GO systems that rely only on nanoparticle distribution, our design exploits a synergistic result: GO enhances photon absorption and scattering at the nanoscale, whereas carbon fabric provides structural strengthening and additional attenuation through π-electron interactions and secondary scattering. This dual mechanism improves shielding effectiveness, reduces thickness necessities, and maintains lightweight flexibility, creating the distinct contribution of this study to gamma-ray shielding analysis. The objective is to establish correlations between GO loading, composite structure, and photon energy, thereby providing a comprehensive framework for the development of next-generation, lightweight radiation shielding materials.

## 2 Materials and methods

### 2.1 Modified Hummer's method

**Fig. 1:**
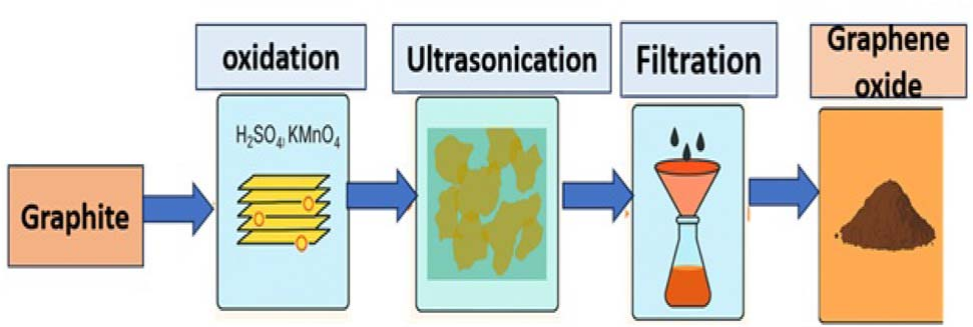
Schematic representation of the synthesis process.

Graphene oxide (GO) nanoparticles were synthesized using a modified Hummers' method followed by thermal calcination as shown by the schematic diagram in Fig. 1. In this process, natural graphite powder was gradually oxidized by introducing it into a mixture of concentrated sulfuric acid (H_2_SO_4_) and phosphoric acid (H_3_PO_4_) under ice-cooled conditions. Potassium permanganate (KMnO_4_) was then added slowly while maintaining the reaction temperature below 10 °C. The mixture was subsequently stirred and heated at 35–40 °C for several hours to ensure complete oxidation. The reaction was terminated by quenching with deionized (DI) water and hydrogen peroxide (H_2_O_2_), resulting in a golden-brown suspension of oxidized graphite. The product was repeatedly washed with hydrochloric acid (HCl) and DI water until a neutral pH was achieved. The obtained GO was separated by centrifugation and dried at 60 °C under vacuum. To further purify and enhance the properties of the material, a calcination step was performed. The dried RGO powder was placed in a muffle furnace and calcined at 400 °C for 4 hours in air or an inert atmosphere. This calcination step partially reduces GO by removing some oxygen-containing functional groups, which enhances its thermal stability and compatibility with the epoxy matrix. However, we note that such partial reduction reduces water dispersibility and can promote some degree of aggregation in aqueous systems. The resulting calcined RGO nanoparticles were collected and subsequently employed as nanofillers in composite preparation (Table 1).

**Table 1:** Composition and physical parameters of the fabricated composites

Sample	% Carbon fabric	% Epoxy	% GO	Thickness of carbon fabric	Thickness of composite	Density (g cm^−3^) GO	Density (g cm^−3^) epoxy
S0	50.00	50.00	0.00	0.2 mm	0.9 mm	1.70	1.16
S1	35.71	62.00	1.93	0.2 mm	0.9 mm	1.70	1.16
S2	31.25	66.69	2.06	0.2 mm	0.9 mm	1.70	1.16
S3	26.32	71.47	2.21	0.2 mm	0.9 mm	1.70	1.16

### 2.2 Hand lay-up method

**Fig. 2:**
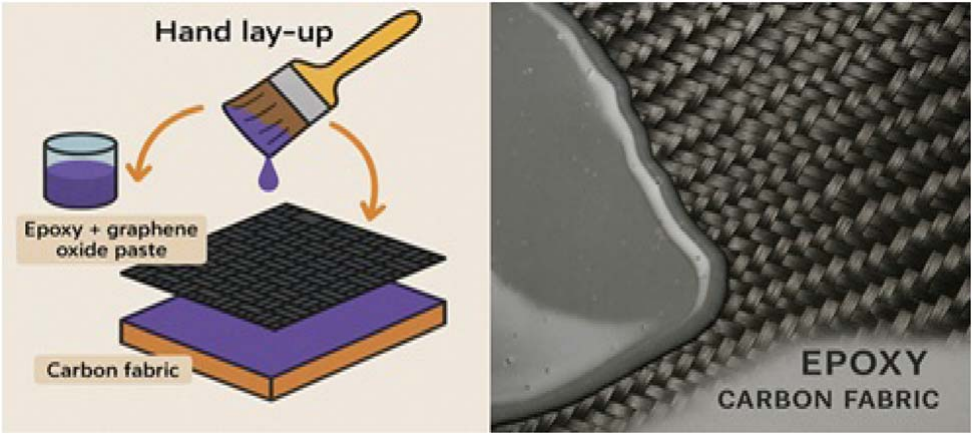
Schematic representation of the hand lay-up method used for the fabrication of a hybrid composite.

The fabrication of the RGO-reinforced epoxy/carbon fabric hybrid composite was performed using the hand lay-up technique as shown in Fig. 2. Initially, acetone was introduced into the epoxy resin to reduce its viscosity and facilitate uniform dispersion. The epoxy acetone mixture was subjected to ultrasonication for 15 minutes using a probe sonicator to enhance solvent resin mixing. Subsequently, a predetermined amount of RGO nanopowder was incorporated into the mixture, followed by mechanical stirring for 20 minutes to achieve preliminary dispersion of the filler. The resulting suspension was further ultrasonicated for an additional 15 minutes to ensure homogeneous dispersion of RGO within the epoxy matrix. After preparation, the RGO/epoxy mixture was manually applied onto layers of woven carbon fabric using the hand lay-up method. Special care was taken to ensure uniform resin distribution and minimize air entrapment. Lastly, the laminated structure was left to cure under ambient conditions for 24 hours to ensure complete crosslinking of the epoxy. A post-curing step was then completed in a hot-air oven at 80 °C for 2 hours, which improved the thermal stability and structural reliability of the composites. The cured samples were consequently demolded and stored at room temperature prior to characterization.

## 3 Results and discussion

### 3.1 XRD spectra of hybrid composite

**Fig. 3:**
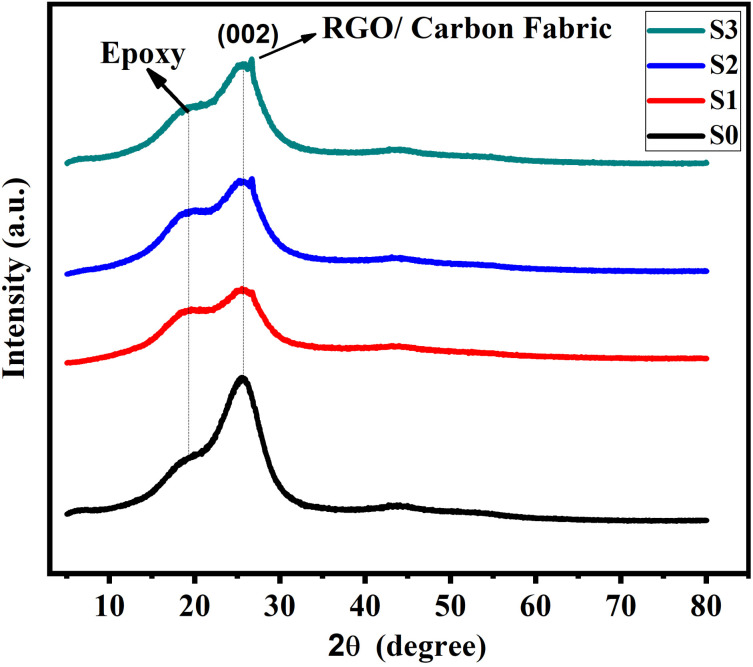
XRD Spectra of epoxy/carbon fabric reinforced with RGO.

X-ray diffraction (XRD) analysis was employed to investigate the phase characteristics and crystallinity of carbon fabric/epoxy composites reinforced with varying amounts of RGO. Fig. 3 presents the diffraction patterns of four samples (S0–S3), each containing carbon fabric but differing in RGO loading from 0 to 5 wt%. In this system, RGO was incorporated as a two-dimensional nanofiller to enhance structural and interfacial properties.^28^ The diffraction pattern of sample S0 (epoxy with 0 wt% RGO) exhibited a broad amorphous peak centered at 2*θ* ≈ 17°, which is characteristic of the disordered crosslinked structure of cured epoxy resin.^29^ Additionally, a low-intensity peak near 2*θ* ≈ 26° was observed, corresponding to the (002) reflection of graphitic carbon, arising from the embedded carbon fabric.^30^ With the progressive incorporation of RGO in samples S1 (3 wt%), S2 (4 wt%), and S3 (5 wt%), the XRD spectra revealed a gradual sharpening and intensification of the (002) peak at 2*θ* ≈ 26°. This behavior reflects enhanced graphitic ordering induced by the presence of RGO nanosheets.^31^ Notably, a slight shift of the (002) peak toward lower 2*θ* values was observed with increasing RGO content. This shift may be attributed to enlarged interlayer spacing between RGO sheets or partial exfoliation/restacking phenomena within the epoxy matrix, consistent with previous reports on GO-based nanocomposites.^32^ The most pronounced (002) reflection was recorded for sample S3 (5 wt% RGO), indicating a synergistic enhancement in graphitic crystallinity, likely resulting from interfacial alignment between RGO nanosheets and carbon fabric filaments. The observed variations in peak intensity and position confirm structural evolution and improved RGO dispersion within the epoxy matrix.^16,33^ These results collectively indicate that the incorporation of RGO nanoparticles as nanofillers not only enhances the visibility of graphitic domains but also induces subtle microstructural rearrangements, confirming the successful synthesis of a hybrid epoxy/carbon fabric/RGO nanocomposite system. However, XRD is not the best tool to determine crystal layer delamination or the homogeneity of dispersion. High magnification electron microscope can be used to confirm the homogeneity of the composites.

### 3.2 FTIR analysis

**Fig. 4:**
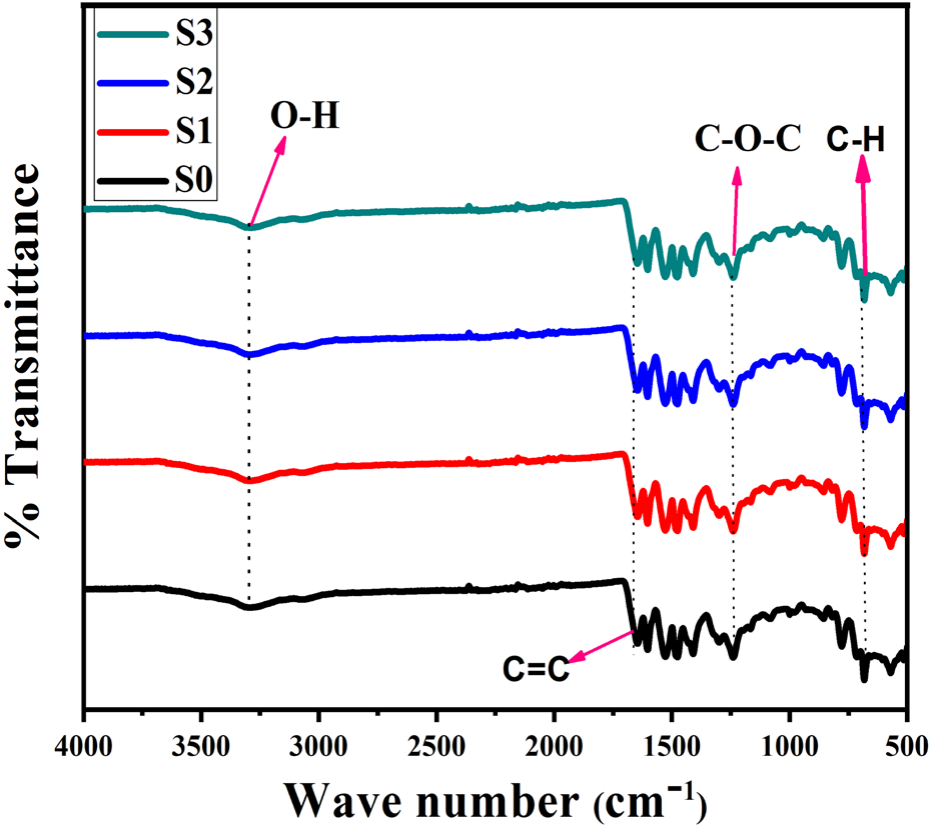
FTIR Spectra of epoxy/carbon fabric reinforced with RGO.

The FTIR spectra of epoxy/carbon fabric composites containing different concentrations of RGO is displayed in Fig. 4. The spectra exhibit characteristic absorption bands associated with the functional groups present in both the epoxy matrix and the RGO nanofillers. A broad absorption band around 3360 cm^−1^, observed in samples S1, S2, and more prominently in S3, corresponds to O–H stretching vibrations, indicating the presence of hydroxyl groups introduced through the RGO nanosheets.^34^ This feature is less pronounced in S0 due to the absence of RGO, thereby confirming the incorporation of oxygen-containing functionalities *via* RGO addition. A distinct band near 1600 cm^−1^, attributed to C=C stretching, is present in all spectra and can be assigned to the aromatic ring vibrations of epoxy resin as well as the conjugated carbon framework of RGO. The absorption band around 1240 cm^−1^ corresponds to C–O–C asymmetric stretching in the epoxy network, while the band near 750 cm^−1^ is associated with C–H out-of-plane bending modes.^35,36^ With increasing RGO concentration from S1 to S3, a slight intensification and shift in the O–H and C–O–C bands were observed, which can be attributed to enhanced interactions between RGO nanosheets and the epoxy matrix through hydrogen bonding and interfacial adhesion. These spectral modifications confirm the formation of hybrid interfacial networks between the functional groups of RGO and epoxy resin.^37,38^ The presence of characteristic epoxy and RGO absorption bands, together with their systematic evolution with increasing RGO content, validates the successful synthesis of epoxy/RGO hybrid composites. These results demonstrate the potential of GO incorporation to tailor the chemical structure and interfacial interactions of carbon fabric-reinforced epoxy composites.

### 3.3 Morphological analysis

**Fig. 5:**
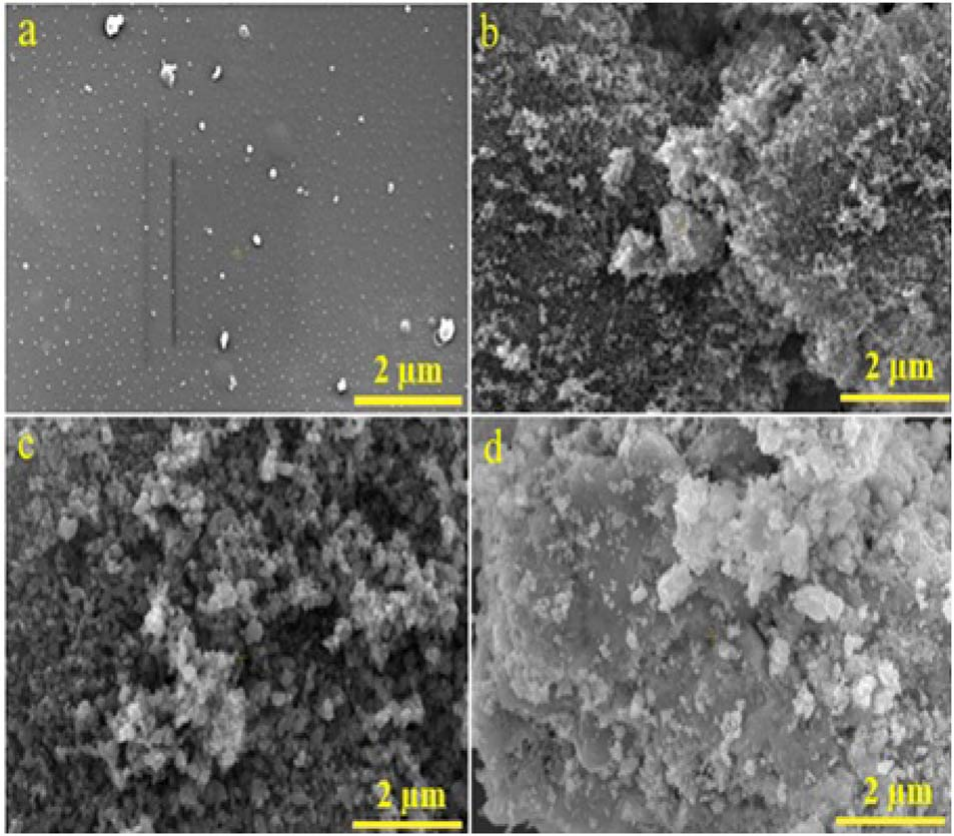
SEM Micrograph (a)–(d) of epoxy/carbon fabric reinforced with RGO.

The SEM micrographs in Fig. 5 illustrate the surface morphology of epoxy/carbon fabric composites reinforced with varying concentrations of RGO. All images were acquired at a magnification of 2464× using an ETD detector under an accelerating voltage of 20.00 kV. Image (a) corresponds to the reference sample (0 wt% RGO), which exhibits a relatively smooth and homogeneous surface with negligible particulate features. This morphology reflects the absence of nanofillers and serves as a baseline for comparison. In contrast, image (b), representing 3 wt% RGO, displays uniformly dispersed bright spots associated with RGO nanosheets embedded within the epoxy matrix. Such uniform dispersion enhances interfacial bonding and indicates improved mechanical and barrier properties. With increased loading to 4 wt% RGO (image c), a higher density of nanoparticles is evident while still maintaining a relatively homogeneous distribution. This concentration appears optimal for achieving effective dispersion without inducing agglomeration.^39–41^ At 5 wt% RGO (image d), however, the microstructure reveals pronounced agglomeration and clustering of RGO particles. Although aggregation can create localized stress concentrations and structural heterogeneity, it simultaneously enhances gamma-ray attenuation due to the higher fraction of high-Z elements and increased interfacial scattering.^42^ Therefore, while excessive RGO loading may compromise microstructural uniformity, it can significantly improve radiation shielding efficiency. This highlights a trade-off between mechanical integrity and attenuation performance, suggesting that higher filler loadings can still be advantageous in applications where shielding effectiveness is prioritized.

### 3.4 Correlation of simulated and experimental results

#### 3.4.1 Mass attenuation coefficients (MAC)

The mass attenuation coefficient (MAC), typically expressed in units of cm^2^ g^−1^, is a fundamental parameter that quantifies the ability of a material to attenuate gamma- or X-ray photons per unit mass.^43^ It represents the probability of interaction between incident photons and the atoms within a material, and depends on both the photon energy and the elemental composition of the medium. The MAC is particularly critical in radiation shielding analysis, as it allows for the comparison of different materials independent of their densities. It is defined by the relation1
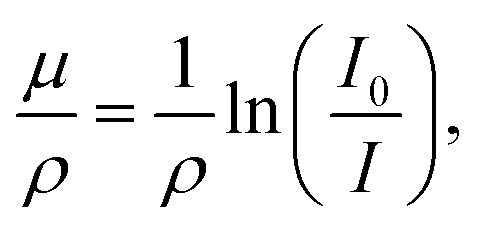
where *μ* is the linear attenuation coefficient, *ρ* is the material density, *I*_0_ is the incident photon intensity, and *I* is the transmitted intensity. Higher MAC values indicate superior shielding efficiency, particularly for materials containing high atomic number (Z) elements that favor photoelectric absorption at lower photon energies.^44,45^ Accurate determination of MAC is therefore essential for the design and optimization of radiation-protective composites and may be obtained experimentally or through computational databases such as XCOM and Phy-X/PSD. In this work, the experimental MAC (*μ*/*ρ*) was determined for the composite samples using a NaI(Tl) scintillation detector and three standard gamma-emitting radionuclide sources: Cs-137 (662 keV), Co-60 (1173 keV and 1332 keV), and Ba-133 (356 keV).^46^ A narrow-beam transmission geometry was adopted to minimize scattering effects.

**Fig. 6:**
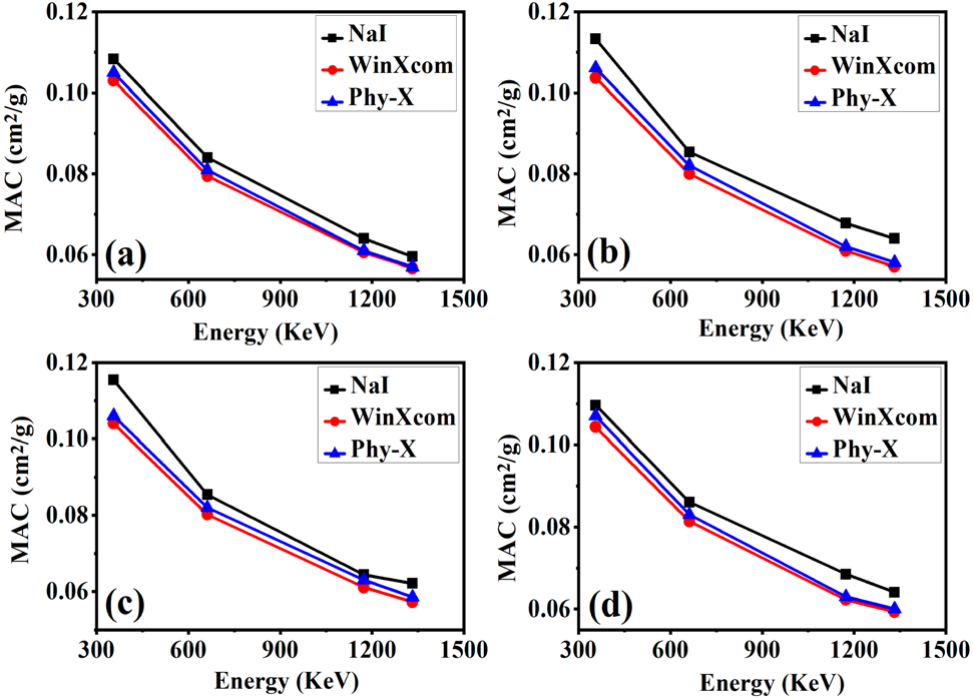
Comparison of gamma-ray attenuation parameter MAC (a)–(d), obtained from experimental measurements using a NaI (Tl) detector and theoretical calculations using Phy-X/PSD and WinXCom for epoxy–carbon fabric-RGO composites.

Fig. 6 presents the variation of MAC as a function of incident photon energy for epoxy/carbon fabric composites reinforced with different RGO contents. The MAC values were obtained from three sources: (i) experimental measurements using a NaI(Tl) detector, (ii) Phy-X/PSD simulations, and (iii) WinXCom calculations. Four composite samples were investigated: S0 (0 wt% RGO), S1 (3 wt% RGO), S2 (4 wt% RGO), and S3 (5 wt% RGO). Across all samples, a decrease in MAC with increasing photon energy is observed, consistent with the transition from photoelectric absorption dominance at lower energies to Compton scattering at higher energies.^47–49^ The inclusion of RGO significantly enhanced the MAC values, attributed to the increased effective atomic number and electron density of the composites. Among all samples, S3 (5 wt% RGO) exhibited the highest MAC across the investigated energy spectrum, indicating superior photon attenuation capacity. This enhancement suggests that further RGO incorporation may eventually reach a saturation limit due to potential filler agglomeration. Importantly, the strong agreement between experimental and theoretical datasets validates the predictive fidelity of WinXCom and Phy-X for evaluating gamma-ray shielding performance. The 5 wt% RGO reinforced epoxy/carbon fabric composite (S3) demonstrates the most promising attenuation characteristics, underscoring its potential as a high-performance candidate for radiation shielding in nuclear, aerospace, and medical applications.

#### 3.4.2 Linear attenuation coefficient (LAC)

The linear attenuation coefficient (*μ*) is a fundamental parameter that characterizes the ability of a material to attenuate gamma radiation per unit thickness (cm^−1^).^50,51^ It quantifies the fraction of a gamma-ray beam that is absorbed or scattered as it traverses a material, without accounting for the influence of material density.^52^ The coefficient is defined by the exponential attenuation law

**Fig. 7:**
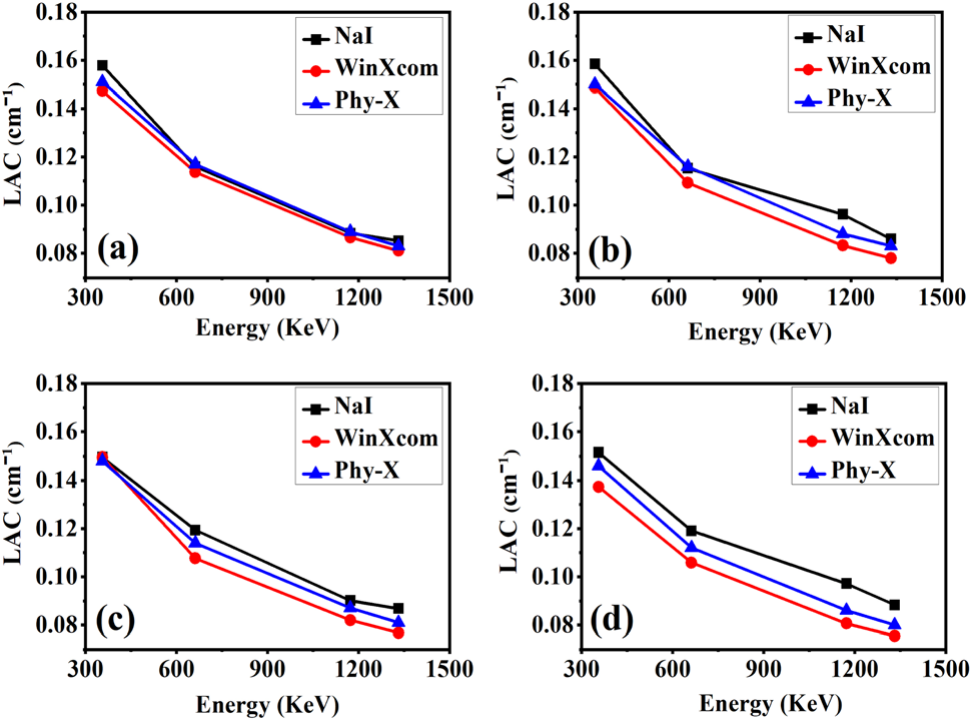
Comparison of gamma-ray attenuation parameter LAC (a)–(d), obtained from experimental measurements using a NaI (Tl) detector and theoretical calculations using Phy-X/PSD and WinXCom for epoxy carbon fabric RGO composites.

2 *I* = *I*_0_*e*^−*μx*^,

where *I*_0_ is the incident photon intensity, *I* is the transmitted photon intensity, *μ* is the linear attenuation coefficient, and *x* is the material thickness. Fig. 7 presents the variation of the linear attenuation coefficient (LAC, cm^−1^) as a function of incident photon energy (356–1332 keV) for epoxy/carbon fabric hybrid composites reinforced with varying reduced graphene oxide (RGO) contents: S0(a), S1(b), S2(c), and S3(d). The LAC values were derived from experimental measurements using a NaI(Tl) detector and cross validated against theoretical predictions from the WinXCom and Phy-X/PSD databases. Across all compositions and methodologies, an inverse dependence of LAC on photon energy is observed. This behavior is consistent with dominant attenuation mechanisms, where photoelectric absorption governs at lower photon energies, while Compton scattering predominates at intermediate to high energies. Incorporation of RGO into the composite matrix significantly enhances the LAC, attributed to the increased effective atomic number and electron density introduced by the nanofiller. Among all samples, S3 (5 wt% RGO) exhibits the highest LAC values, particularly at low to intermediate photon energies (356–662 keV), confirming its superior gamma attenuation capability. The close agreement between experimental data and theoretical simulations highlights the robustness of predictive computational frameworks for photon matter interactions in polymeric shielding systems. These results demonstrate that RGO incorporation up to 5 wt% optimally improves the radiation shielding efficiency of epoxy/carbon fabric composites, establishing S3 as a promising candidate for advanced gamma-ray protective materials.

#### 3.4.3 Half value layer (HVL)

The half value layer (HVL) is a fundamental shielding parameter that defines the thickness of a material required to reduce the intensity of incident gamma radiation by 50%. It is mathematically related to the linear attenuation coefficient (*μ*) as3
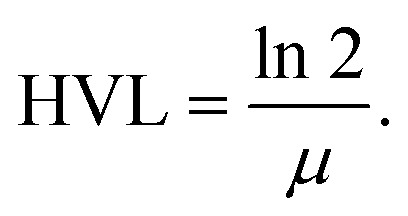


**Fig. 8:**
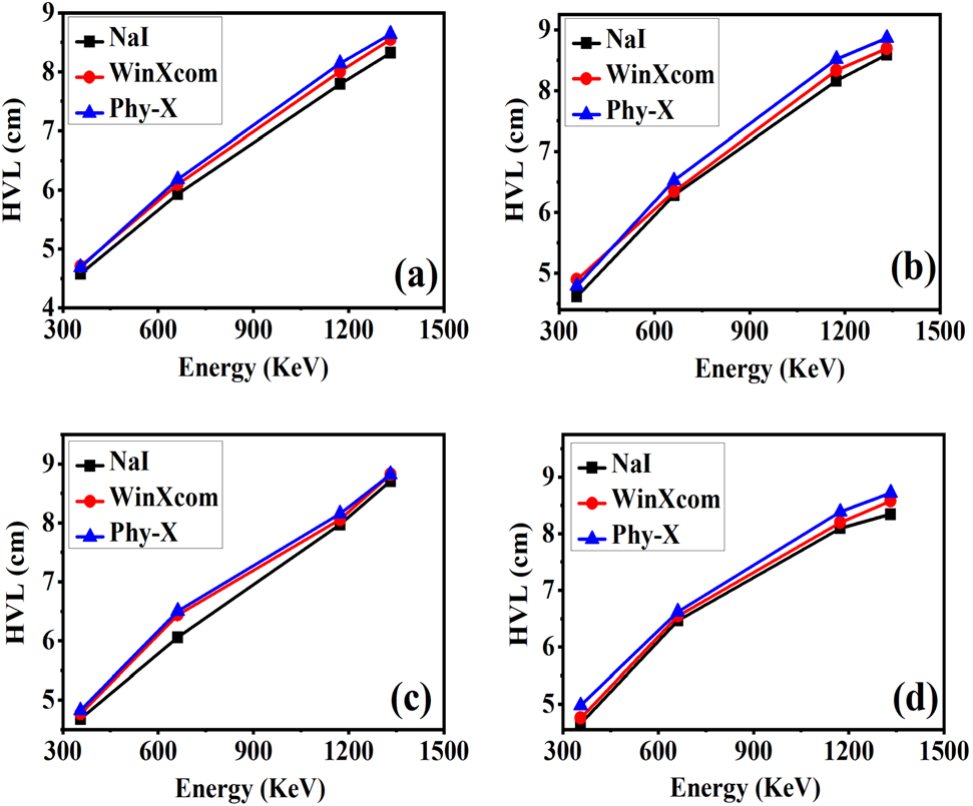
Comparison of gamma-ray attenuation parameter HVL (a)–(d), obtained from experimental measurements using a NaI (Tl) detector and theoretical calculations using Phy-X/PSD and WinXCom for epoxy carbon fabric RGO composites.

Lower HVL values indicate superior shielding efficiency, as less material is needed to attenuate the radiation.^53^ HVL is strongly energy-dependent: at lower photon energies, the photoelectric effect dominates, resulting in higher attenuation and consequently lower HVL values.^54^ Conversely, at higher photon energies, Compton scattering becomes the predominant mechanism, which reduces photon–matter interaction probability and leads to higher HVL values.^55^Fig. 8 illustrates the variation of HVL with photon energy (356–1332 keV) for epoxy/carbon fabric hybrid composites reinforced with different RGO concentrations. The HVL values were obtained experimentally using a NaI(Tl) detector and validated through theoretical calculations using the Phy-X/PSD and WinXCom databases. Across all samples, HVL is observed to increase monotonically with photon energy, consistent with the inverse dependence of HVL on the linear attenuation coefficient (LAC). At higher energies, where LAC values decrease, a greater thickness is required to achieve 50% photon attenuation.^56^

Among the studied composites, S3 (5 wt% RGO) consistently exhibits the highest HVL values. This suggests that although increased RGO loading enhances photon interaction, excessive filler concentration may induce agglomeration or non-uniform dispersion, limiting further shielding improvement beyond the 5 wt% threshold. In contrast, S1 (3 wt% RGO) and S2 (4 wt% RGO) demonstrate moderate HVL values and more favorable attenuation performance compared to the control sample S0 (0 wt% RGO). This indicates that intermediate RGO concentrations promote optimal matrix–filler interaction and effective dispersion of nanofillers. The excellent agreement between experimental and theoretical HVL values validates the predictive robustness of WinXCom and Phy-X/PSD models for gamma shielding analysis. Overall, these findings emphasize the importance of optimizing RGO content to balance microstructural uniformity and photon attenuation efficiency. Notably, the S3 composite demonstrates the best overall performance, highlighting its potential application in the design of lightweight and flexible radiation shielding materials for nuclear medicine, aerospace, and defense technologies.^57,58^

#### 3.4.4 Tenth value layer (TVL)

The tenth value layer (TVL) represents the thickness of a material required to reduce the intensity of incident gamma radiation by 90%.^59^ It is mathematically related to the linear attenuation coefficient (*μ*) as4
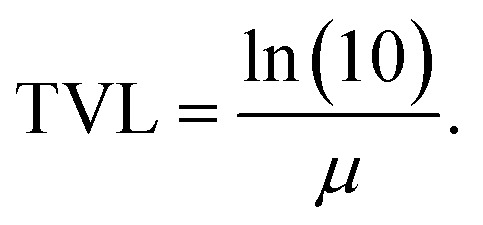


**Fig. 9:**
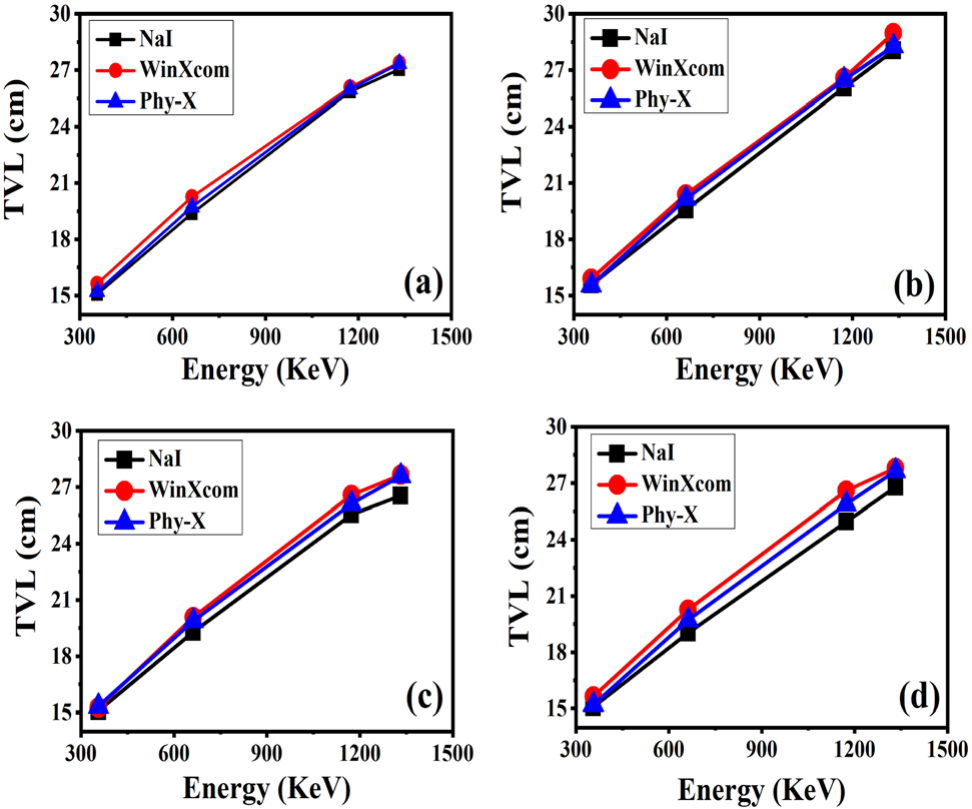
Comparison of gamma-ray attenuation parameter TVL (a)–(d), obtained from experimental measurements using a NaI(Tl) detector and theoretical calculations using Phy-X/PSD and WinXCom for epoxy carbon fabric RGO composites.

Compared to the half value layer (HVL), the TVL provides a more stringent criterion for shielding performance, making it particularly relevant in environments demanding high attenuation, such as nuclear medicine, reactor shielding, and aerospace applications.^60^Fig. 9 illustrates the variation of TVL with photon energy (356–1332 keV) for epoxy/carbon fabric hybrid composites containing different weight fractions of RGO. The values were determined experimentally using a NaI(Tl) scintillation detector and validated through theoretical predictions obtained from WinXCom and Phy-X/PSD databases. Across all samples, TVL is observed to increase with rising photon energy, which is consistent with the reduced attenuation efficiency at higher energies where Compton scattering dominates over photoelectric absorption.^61–64^

Among the investigated composites, the control sample S0 (0 wt% RGO) consistently exhibits the highest TVL values across the entire energy range, signifying its relatively weak shielding capability. In contrast, the incorporation of RGO markedly reduces TVL, reflecting improved attenuation efficiency due to the enhanced effective atomic number and electron density of the nanofiller-reinforced matrix.^65^ Notably, sample S3 (5 wt% RGO) demonstrates the lowest TVL values at all photon energies, thereby confirming its superior shielding efficiency. This improvement is attributed to the uniform dispersion of RGO nanosheets within the epoxy matrix, which enhances both scattering and absorption interactions with incident photons.^66,67^ The strong agreement between experimental and simulated TVL values validates the reliability of both WinXCom and Phy-X/PSD methodologies. Overall, these findings identify S3 as the most effective shielding formulation, underscoring its potential for practical use in advanced radiation protection systems, including diagnostic imaging, nuclear safety barriers, and aerospace shielding structures.

#### 3.4.5 Mean free path (MFP)

The mean free path (MFP) is a fundamental parameter in radiation shielding, representing the average distance a gamma-ray photon travels within a material before undergoing an interaction such as photoelectric absorption or Compton scattering. Mathematically, the MFP is inversely proportional to the linear attenuation coefficient (*μ*), and is expressed as5
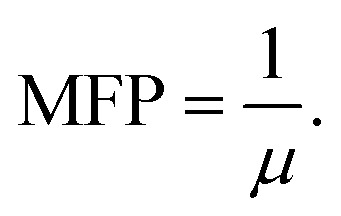


**Fig. 10:**
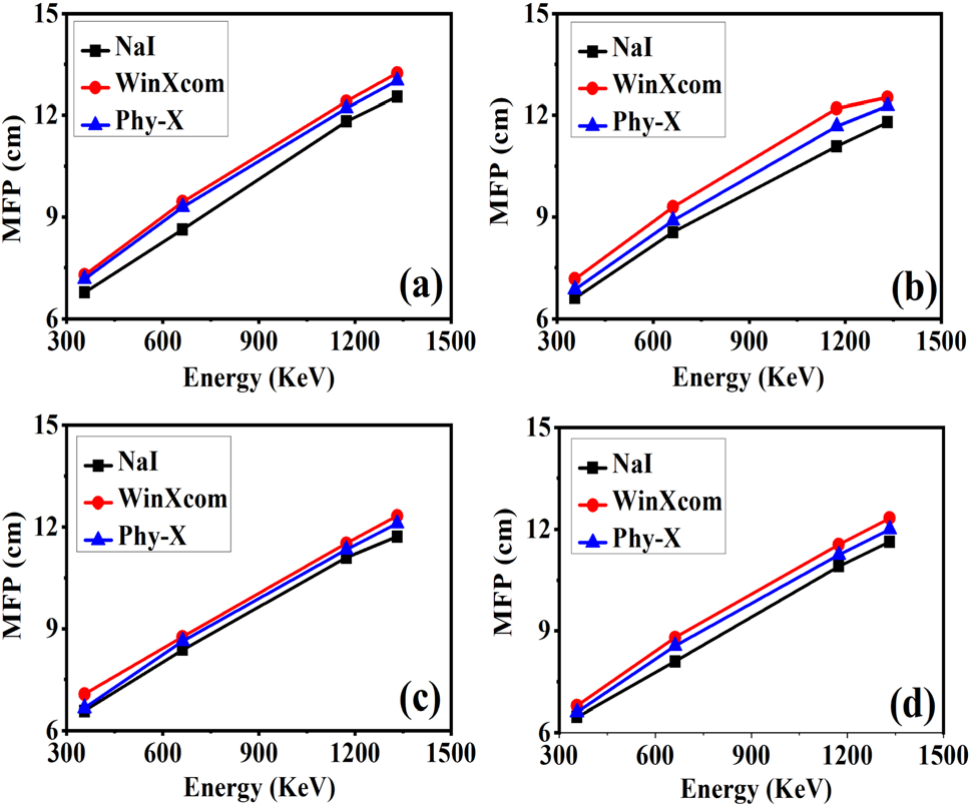
Comparison of gamma-ray attenuation parameter MFP (a)–(d), obtained from experimental measurements using a NaI(Tl) detector and theoretical calculations using Phy-X/PSD and WinXCom for epoxy carbon fabric RGO composites.

Fig. 10 illustrates the variation of MFP values for gamma photons in composite samples, obtained using three approaches: experimental NaI(Tl) detector-based measurements and theoretical simulations *via* WinXCom and Phy-X/PSD, as a function of photon energy in the range 356–1332 keV. In all cases, a consistent trend of increasing MFP with photon energy is observed, which is expected due to the reduced probability of photon matter interaction at higher energies.^71^ Among the three methods, the experimental data generally yield slightly lower MFP values compared to theoretical predictions. This deviation may be attributed to microstructural heterogeneities, imperfections, or additional attenuation mechanisms present in real samples but not captured in theoretical models. WinXCom and Phy-X results exhibit close agreement across all energies, with Phy-X occasionally predicting marginally lower values due to its updated cross-section databases and flexible compositional inputs. The strong correlation between experimental and simulated values validates the predictive reliability of these computational tools, confirming their usefulness for rapid screening and design of shielding materials.^72^ Among the investigated composites, sample S3 [Fig. 10d] consistently demonstrates the lowest MFP across all energy levels, indicating the highest photon interaction probability and, consequently, superior shielding performance. This suggests that S3, with optimal RGO incorporation, represents the most effective gamma-shielding composition in the series.

Table 2 summarizes a comparison between the present study and recently reported works on gamma-ray shielding materials. While most prior studies have focused on glass- or geopolymer-based systems incorporating high-Z fillers such as lead, bismuth oxide, or rare-earth additives, these materials often suffer from drawbacks including brittleness, high density, or toxicity. For example, Pb-doped geopolymers enhance attenuation efficiency but increase weight and pose health risks, whereas Bi_2_O_3_-based glasses provide good shielding yet remain dense and fragile. Similarly, epoxy composites with mixed metallic fillers show improvements mainly in the X-ray energy regime, but lack validation in the high-energy gamma range. In contrast, the present work introduces a lightweight epoxy/carbon fabric hybrid reinforced with graphene oxide, which not only reduces thickness demand but also achieves superior mass and linear attenuation coefficients validated against experimental and simulation methods. This highlights the novelty of combining fabric reinforcement with RGO fillers to achieve effective, flexible, and non-toxic gamma-ray shielding solutions.

**Table 2:** Comparison of gamma-ray shielding studies with the present work

Study	Material system	High-Z filler(s)	Methods	Novelty/limitation	This Work's
This work	Epoxy + woven carbon fabric	Graphene oxide (3–5 wt%)	Experimental with NaI(Tl) at 356, 662, 1173, 1332 keV; validated using Phy-X/PSD and WinXCom	5 wt% RGO sample shows best MAC/LAC and lowest TVL across energies	Lightweight hybrid with fabric reinforcement; superior attenuation with reduced thickness demand
*Sci. Rep.* (2023)^67^	Alkali-activated geopolymer (GEO-*x*Pb)	Pb additive (10–20 wt%)	XCOM/WinXCom MAC and measured density; *μ* and HVL at ∼0.6 MeV	Pb addition improves *μ* (*e.g.*, 0.252 cm^−1^ at 0.6 MeV); but material is heavy and toxic	Lead-free, lightweight composite achieving strong attenuation in medical isotope energy range
*Pamukkale Univ. J. Eng. Sci.* (2022)^68^	BaO–B_2_O_3_–TeO_2_ (BBT) glass	Bi_2_O_3_, Gd_2_O_3_, La_2_O_3_, Sm_2_O_3_ (2.5 mol%)	HVL reported at 0.662 MeV; shielding efficiency per cm derived	Bi_2_O_3_-doped glass shows HVL ≈ 1.67 cm (∼34%/cm); brittle and dense	Polymer composite offers flexibility and lower density while maintaining competitive attenuation
*Polymers* (2025)^69^	Epoxy composites	Mixed metallic/oxide fillers (Bi_2_O_3_, Ta, BaSO_4_, *etc.*)	Experimental attenuation in X-ray range (20–120 keV)	MAC improvement up to ∼100% in X-ray regime; no validation at 0.662–1.33 MeV	Direct validation at Cs-137 and Co-60 gamma energies using experiment + simulation
*J. Radiat. Res. Appl. Sci.* (2025)^70^	CaO–B_2_O_3_–SiO_2_ (CBSi-*x*) glass	(Composition-driven *Z*_eff_)	Provides *μ*/*ρ vs.* energy (0.015–15 MeV) and *Z*_eff_; no HVL at 0.662 MeV	Trends are informative, but lack explicit HVL for shielding per-cm analysis	Full experimental validation with HVL/TVL trends across standard gamma energies

## 4 Conclusions

In this study, epoxy/carbon fabric composites reinforced with varying concentrations of reduced graphene oxide (RGO) were synthesized and characterized for structural and gamma-ray shielding properties. RGO incorporation enhanced attenuation efficiency, reflected in higher MAC and LAC values and reduced HVL and MFP. The 5 wt% RGO composite (S3) showed the best shielding performance despite some SEM-observed aggregation, as increased effective atomic number and scattering centers outweighed dispersion limitations; future TEM studies could provide deeper nanoscale insight. FTIR and XRD confirmed successful RGO integration, while the synergistic effect of RGO nanosheets and carbon fabric reinforcement yielded superior attenuation. Beyond shielding, the hybrid structure is also expected to improve mechanical strength through better load transfer and interfacial adhesion, underscoring the multifunctional potential of these composites. These findings establish epoxy/carbon fabric–RGO hybrids as lightweight, efficient candidates for next-generation radiation shielding, with future work directed toward optimizing dispersion, long-term durability, and application-specific evaluations.

## Author contributions

Summan Urooje: conceptualization, methodology, experimental work, data curation, formal analysis, writing original draft. Ahsan Irshad: software, computational simulations, writing, review & editing. Danish Arif: software, computational simulations, writing, review & Editing. Akif Safeen: supervision, resources, project administration, validation. Basit Ali: review & editing, resources. All authors reviewed and approved the final manuscript.

## Conflicts of interest

There are no conflicts to declare.

## Data Availability

Data will be available upon request.
